# Common genetic variants associated with obesity in an African-American and Hispanic/Latino population

**DOI:** 10.1371/journal.pone.0250697

**Published:** 2021-05-13

**Authors:** Brandon Chalazan, Denada Palm, Arvind Sridhar, Christina Lee, Maria Argos, Martha Daviglus, Jalees Rehman, Sreenivas Konda, Dawood Darbar

**Affiliations:** 1 Department of Medicine, University of Illinois at Chicago, Chicago, Illinois, United States of America; 2 Broad Institute of MIT and Harvard, Boston, Massachusetts, United States of America; 3 Division of Epidemiology and Biostatics, School of Public Health, University of Illinois at Chicago, Chicago, Illinois, United States of America; 4 Institute for Minority Health Research, University of Illinois at Chicago, Chicago, Illinois, United States of America; 5 Department of Pharmacology, University of Illinois at Chicago, Chicago, Illinois, United States of America; GeneDx, UNITED STATES

## Abstract

**Introduction:**

Over 35% of all adults in the world are currently obese and risk of obesity in racial or ethnic minority groups exist in the US, but the causes of these differences are not all known. As obesity is a leading cause of cardiovascular disease, an improved understanding of risk factors across racial and ethnic groups may improve outcomes.

**Objective:**

The objective of this study was to determine if susceptibility to obesity is associated with genetic variation in candidate single nucleotide polymorphisms (SNPs) in African Americans and Hispanic/Latinos.

**Materials and methods:**

We examined data from 534 African Americans and 557 Hispanic/Latinos participants from the UIC Cohort of Patients, Family and Friends. Participants were genotyped for the top 26 obesity-associated SNPs within *FTO*, *MC4R*, *TUB*, *APOA2*, *APOA5*, *ADIPOQ*, *ARL15*, *CDH13*, *KNG1*, *LEPR*, *leptin*, and *SCG3* genes.

**Results:**

The mean (SD) age of participants was 49±13 years, 55% were female, and mean body mass index (BMI) was 31±7.5 kg/m^2^. After adjusting for age and sex, we found that rs8050136 in *FTO* (odds ratio [OR] 1.40, 95% confidence interval [CI] 1.1–1.8; P = 0.01) among African Americans and rs2272383 in *TUB* (OR 1.34, 95% CI 1.04–1.71; P = 0.02) among Hispanic/Latinos were associated with obesity. However, none of the SNPs in multivariable analysis of either AA or H/L cohorts were significant when adjusted for multiple correction.

**Conclusions:**

We show that candidate SNPs in the *FTO* and *TUB* genes are associated with obesity in African Americans and Hispanic/Latinos individuals respectively. While the underlying pathophysiological mechanisms by which common genetic variants cause obesity remain unclear, we have identified novel therapeutic targets across racial and ethnic groups.

## Introduction

It is estimated that over 35% of all adults in the world are currently obese with a greater prevalence in ethnic minority groups in the US, but the causes of these differences are not all known [1]. According to national data, the prevalence of obesity among the non-Hispanic black population was 47% versus 38% in the non-Hispanic white population [2]. As obesity is a leading cause of cardiovascular disease, an improved understanding of risk factors across racial and ethnic groups may improve outcomes. Although many traditional risk factors for cardiovascular disease have been identified in whites of European descent, ethnicity-specific risk factors are not well defined, especially among H/L individuals in the United States.

The phenotype for obesity is complex and is known to be influenced by socioeconomic, environmental and genetic determinants. Genome-wide association studies (GWAS) have identified common genetic variants associated with obesity [3–6]. Many single nucleotide polymorphisms (SNPs) reside near genes whose role in obesity remains unclear [7]. Despite some evidence in transethnic populations, the genetic architecture of obesity in minority populations largely remains poorly understood. Furthermore, the effects of combining these at-risk SNPs have yet to be fully explored. The aim of our study was to explore the association of candidate obesity SNPs among African American (AA) and Hispanic/Latino (H/L) individuals to better understand how these common genetic variations contribute to the risk of obesity across multi-ethnic populations.

## Materials and methods

### Ethics statement

We confirm that the University of Illinois at Chicago (UIC) Institutional Review Board specifically approved this study.

### Study population

We used a random subset of AA and H/L participants from the UIC Cohort of Patients, Family and Friends (UIC Cohort). Selected participants self-identified as being AA or H/L and were aged 18 years or older. The sample for the present analysis consisted of 1,091 participants, of which ~30% were obese (body mass index [BMI] ≥30 kg/m^2^). Information on demographic characteristics, risk factors, and medical history, and blood for DNA extraction were obtained at the enrollment visit using a detailed questionnaire. All participants had height and weight measured by a trained study interviewer at the enrollment visit, from which BMI was derived and categorized into obese (BMI≥30 kg/m^2^) or non-obese (BMI<30 kg/m^2^) groups for the purposes of these analyses.

### Definitions

Obesity was defined as a BMI ≥ 30 kg/m^2^ and non-obese participants had a BMI < 30 kg/m^2^, based on measured height and weight at the time of enrollment. Arterial hypertension (HTN) was defined by a history of HTN and/or the presence of antihypertensive therapy. Criteria for coronary artery disease (CAD) included a history of myocardial infarction (MI), previous coronary bypass surgery or percutaneous coronary intervention (PCI). Congestive heart failure (CHF) was defined by a history of CHF. A history of obstructive sleep apnea (OSA) was considered positive if the participant had a positive sleep study or was receiving continuous positive airways pressure therapy. Left atria (LA) and left ventricular (LV) measurements from the M-mode echocardiograms were done by an experienced cardiologist.

### Sample processing

DNA extraction from the buffy coat layer containing the white blood cells was conducted from the collected serum. DNA was extracted using a commercially available kit (Qiagen Puregene, Valencia, CA), and samples were stored in -80°C until genotyping. Samples were processed according to the Agena iPLEX Gold Protocol. DNA concentrations were between 5–200 ng/μl and aliquots of 2 μl were transferred to a 384 well reaction plate for the target specific multiplex PCR amplification. This was followed by shrimp alkaline phosphatase (SAP) treatment, and then a single base extension which was carried out for each multiplex plate. The reaction products were transferred to SpectroCHIP arrays using the RS1000 Nanodispenser. The spectral analysis for single bases when incorporated during extension steps was completed using Matrix Assisted Laser Desorption Ionization—Time of Flight (MALDI-TOF) mass spectrometer and MassARRAY Analyzer software.

### Genetic variants

We selected SNPs in the following candidate genes: *FTO*, *MC4R*, *TUB*, *APOA2*, *APOA5*, *ADIPOQ*, *ARL15*, *CDH13*, *KNG1*, *LEPR*, *leptin*, and *SCG3* that have been significantly associated with obesity in whites of European descent identified in genome wide association studies (GWAS) [3–11]. Common genetic variants in the fat mass and obesity-associated (*FTO*) locus have consistently been associated with obesity in mostly whites of European descent with the strongest signal residing in the first and second intronic regions of FTO [12–14]. *RPGRIP1L*, the closest neighboring gene to the FTO locus, is postulated to regulate trafficking of leptin receptors in cilia to maintain the ciliary function in the body with defects in this process causing obesity [15]. Melanocortin-4 receptor (MC4R) is a key regulator of body weight and genetic mutations in *MC4R* gain weight from early childhood [16]. TUB (TUB bipartite transcription factor) is a protein coding gene and has been associated with obesity [6]. A functional SNP in apolipoprotein A-II (*APOA5*) gene promoter has been associated with food intake and obesity risk in non-Hispanic white Americans [3, 10, 11]. The *APOA5* gene encodes apolipoprotein A-V protein which determines plasma triglyceride levels, a major risk factor for obesity. *ADIPOQ* encodes adiponectin, a protein hormone important in regulating glucose levels as well as fatty acid breakdown. ADP-ribosylation factor-like 15 (ARL15) is a protein in humans that is encoded by the *ARL15* gene with a GWAS reporting that variants in *ARL15* not only influenced adiponectin levels but were associated with obesity [17]. A GWAS not only showed that plasma adiponectin levels correlated with the CDH13 locus, which encodes a receptor for high molecular weight forms of adiponectin, but also identified a novel *KNG1*-*ADIPOQ* haplotype that was strongly associated with adiponectin levels in Filipinos [18]. *LEP and LEPR* genes encode leptin and leptin receptor respectively, which are intimately involved in body weight regulation and SNPs in both have been associated with obesity. A SNP in secretogranin III (*SCG3*) was significantly associated with obesity in a Japanese population [11].

Multiplex assay design for genotyping at the 26 SNPs was carried out by Agena Assay Design Suite v2.0. Genotyping calls were made by TyperAnalyzer software using the Autocluster method. Calls were manually reviewed with quality control (QC) and genotype reports in a tabular formation. All SNP calls were >95% with an average call rate of 99%. Furthermore, a polygenic risk score (PRS), consisting of the 26 candidate SNPs for obesity, was constructed by summing the risk alleles weighted by the effect sizes from the GWAS results. Patients were then stratified into quartiles of low: 0–20%; intermediate: 20–80%; and high: 80–100% genetic risk, and the risk of obesity was estimated by a logistic regression model with adjusted sex, age and clinical risk factors (HTN, DM, MI, CHF).

### Statistical methods

Descriptive statistics are presented as mean ± standard deviation (SD) for continuous variables and counts (proportions) for categorical variables. We assessed differences between non-obese controls and obese cases using a chi-square test or a Student’s t-test for variables listed in Tables 1 and 2. Fisher’s exact test was conducted in place of chi-square test when at least one of the expected sample counts was less than 5. Genotyping data, assay statistics, and QC parameters for the selected samples were derived from peak area data. All quality matrices including statistics of assay and sample performance across the entire dataset were used to exclude poor performing samples and assays from downstream analyses. Individual genotype outputs in a tabular format were combined into a large matrix format for subsequent genetic analysis using PLINK and Golden Helix genome data analyses. Using logistic regression in PLINK, association analysis was performed in both unadjusted and adjusted for age and sex assuming an additive genetic model for all SNPs that passed QC. Multiple comparisons were accounted for using the Bonferroni correction (0.05/26 = 0.002) to determine the appropriate significant threshold.

**Table 1:** Selected characteristics of the African American and Hispanic/Latino cohorts.

Characteristics	African American			Hispanic/Latino		
	Non-Obese (BMI<30) n = 274	Obese (BMI≥30) n = 260	P-value	Non-Obese (BMI<30) n = 294	Obese (BMI≥30) n = 263	P-value
Age (SD)	53±11	51±10	0.06	45±14	46±13	0.29
BMI kg/m^2^ (SD)	25±3	37±6	<0.001	26±6	37±6	<0.001
Women (%)	101 (36.0)	173 (66.0)	<0.001	152 (52.0)	174 (66.0)	<0.001
COPD (%)	127 (8.2)	13 (5.0)	0.15	12 (4.2)	7 (2.7)	0.35
Hypertension (%)	172 (46.0)	165 (64.0)	<0.001	75 (25.5)	93 (35.6)	0.01
Diabetes mellitus (%)	35 (13.0)	67 (26.0)	<0.001	2 (0.8)	60 (20.8)	<0.001
RHD (%)	5 (2.0)	7 (2.6)	0.64	5 (0.8)	2 (0.7)	0.90
MI (%)	13 (4.8)	17 (6.6)	0.37	14 (4.8)	6 (2.3)	0.11
Heart failure (%)	21 (8.1)	5 (1.8)	<0.001	4 (1.4)	4 (1.5)	0.89
Stroke (%)	19 (7.0)	15 (5.8)	0.56	8 (2.7)	4 (1.5)	0.34

**Abbreviations**: BMI: Body mass index; COPD: Chronic obstructive pulmonary disease; MI, myocardial infarction; RHD: Rheumatic heart disease. Heart failure for Congestive heart failure.

**Table 2:** Multivariable analysis of BMI groups in African Americans.

CHR	Genes	SNP	Minor Allele	MAF	Adjusted OR*	SE	L95	U95	P-Value
1	APOA2	rs50820000	G	0.245388	1.27	0.15	0.95	1.70	0.10
1	APOA2	rs50850000	G	0.093461	0.89	0.22	0.58	1.36	0.59
1	LEPR	rs11371010	A	0.479325	1.01	0.13	0.77	1.30	0.97
1	APOA2	rs64134530	A	0.024297	0.74	0.43	0.32	1.72	0.49
3	KNG1	rs11924390	C	0.492602	1.24	0.12	0.97	1.59	0.08
3	KNG1	rs18516650	G	0.438151	1.27	0.12	0.99	1.64	0.06
3	ADIPOQ	rs64441750	A	0.314015	1.10	0.14	0.84	1.46	0.45
3	CDH13	rs86426500	T	0.151891	1.23	0.18	0.86	1.76	0.26
5	ADIPOQ	rs43113940	G	0.301842	1.01	0.14	0.77	1.33	0.93
7	Leptin	rs77990390	A	0.091181	1.21	0.30	0.77	1.91	0.40
11	TUB	rs15281330	G	0.147654	1.00	0.18	0.69	1.44	1.00
11	TUB	rs22723830	A	0.129245	1.25	0.19	0.86	1.80	0.24
11	APOA5	rs66279900	G	0.123607	0.81	0.19	0.56	1.20	0.30
15	SCG3	rs16964465	C	0.180158	1.20	0.16	0.88	1.67	0.25
15	SCG3	rs16964476	G	0.182862	1.17	0.16	0.84	1.63	0.35
15	SCG3	rs37642200	G	0.176916	1.23	0.17	0.89	1.71	0.21
16	FTO	rs11219800	A	0.471949	1.27	0.13	0.99	1.64	0.06
16	CDH13	rs38651880	T	0.381064	1.01	0.13	0.79	1.305	0.91
16	ADIPOQ	rs71937880	G	0.1375	0.69	0.20	0.47	1.03	0.07
16	FTO	rs11076023	A	0.334827	0.90	0.14	0.69	1.18	0.45
16	FTO	rs14210850	C	0.084113	1.452	0.23	0.91	2.30	0.11
16	FTO	rs15589020	A	0.074835	1.25	0.24	0.77	2.01	0.37
**16**	FTO	**rs80501360**	**A**	**0.44015**	**1.40**	**0.13**	**1.08**	**1.80**	**0.01**
18	MC4R	rs22296160	T	0.022479	0.67	0.45	0.28	1.64	0.39

**Abbreviations**: CHR, chromosome; SNP, single nucleotide polymorphism; MAF, minor allele frequency; OR, odds ratio; SE, standard error.

* Adjusted for sex and age.

## Results

We examined data from 534 AA and 557 H/L participants from the UIC Cohort. The mean (SD) age of participants was 49±13 years, 55% were female, and the mean BMI was 31±7.5 kg/m^2^. Baseline clinical characteristics and co-morbidities of AA and H/L respectively are shown in Table 1. Two-thirds of the participants were women in both obese groups. As expected the prevalence of HTN and DM were significantly different in the obese and non-obese groups in AAs and H/Ls as was CHF.

Only two SNPs out of 26 failed to meet the Hardy-Weinberg equilibrium (HWE) in AAs (rs26671700, P = 0.02; rs99305060, P = 0.001). Three SNPs rs16964465 (P = 0.0004), rs16964476 (P = 0.002), and rs37642200 (P = 0.002) failed to meet the HWE in H/Ls. The SNPs which did not meet HWE were excluded from multivariable analysis. Thereby, association analysis was performed in 26 candidate SNPs. The genome-wide association permutation tests, a robust measure for those SNPs that failed to meet HWE, showed that neither of these SNPs were significant in either of the two ethnic groups. The same permutation tests resulted in one significant SNP rs8050130 (P = 0.004) in the AA cohort and one rs2272383 with P = 0.02 in the H/L cohort.

The bivariate analysis of 26 SNPs in the obese and non-obese groups in the AA cohort identified one SNP on chromosome 11: rs11219800 (P = 0.03, odds ratio [OR] 1.30, 95% confidence interval [CI] 1.02–1.66; P = 0.02) and two SNPs on chromosome 16: rs71937880 (P = 0.02, OR 0.65, 95% CI 0.0.46–0.94; P = 0.02) and rs8050136 (P = 0.003, OR = 1.44, 95% CI 1.13–1.84; P = 0.003. A similar analysis in the H/L cohort identified one rs2272383 SNP on chromosome 11 (OR = 1.34, 95% CI 1.05–1.72; P = 0.02).

Multivariable analysis in the AA cohort revealed that the rs80501360 SNP on chromosome 16 in *FTO* was associated with obesity (OR 1.40; 95% CI 1.08–1.86; P = 0.01; Table 2). A similar analysis in the H/L cohort identified the rs227383 SNP on chromosome 11 in *TUB* to be associated with obesity (OR 1.34; 95% CI: 1.04–1.71; P = 0.02; Table 3). Analysis of BMI as a quantitative trait, instead of binary non-obese and obese groups, with the 26 candidate SNPs revealed similar results. In the AA cohort, rs8050136 on chromosome 16 was the most significant SNP (P = 0.002) as with the binary analysis. Two additional SNPs rs864265 (P = 0.01) and rs11924390 (P = 0.04) were also associated with a continuous BMI outcome. In the H/L cohort, the rs22772383 remained significant (P = 0.01) as in the binary obese data analysis. One additional SNP rs1558902 was associated (P = 0.04) with the continuous BMI outcome. However, none of the SNPs in multivariable analysis of either AA or H/L cohorts were significant at the Bonferroni corrected alpha 0.05/26 (≈0.002).

**Table 3:** Multivariable analysis of BMI groups in Hispanics/Latinos.

CHR	Genes	SNP	Minor Allele	MAF	Adjusted OR*	SE	L95	U95	P-Value
1	APOA2	rs50820000	G	0.225348	1.2879	0.14	0.97	1.70	0.08
1	APOA2	rs50850000	G	0.236071	0.83	0.15	0.63	1.12	0.23
1	LEPR	rs11371010	G	0.446475	1.09	0.12	0.85	1.38	0.50
1	APOA2	rs64134530	A	0.081824	0.86	0.23	0.55	1.36	0.53
3	KNG1	rs11924390	C	0.320317	0.93	0.13	0.72	1.21	0.61
3	KNG1	rs18516650	G	0.310926	0.86	0.14	0.66	1.13	0.27
3	ADIPOQ	rs26671700	T	0.439887	0.96	0.12	0.76	1.22	0.74
3	ADIPOQ	rs64441750	A	0.301615	0.93	0.13	0.72	1.21	0.60
3	CDH13	rs86426500	T	0.136175	1.03	0.18	0.73	1.46	0.86
5	ADIPOQ	rs43113940	G	0.224208	1.02	0.15	0.76	1.37	0.87
7	Leptin	rs77990390	A	0.349158	1.03	0.12	0.80	1.31	0.83
11	TUB	rs15281330	G	0.140965	0.85	0.17	0.60	1.20	0.35
**11**	TUB	**rs22723830**	**A**	**0.355708**	**1.34**	**0.13**	**1.04**	**1.71**	**0.02**
11	APOA5	rs66279900	G	0.162497	0.92	0.16	0.66	1.27	0.61
16	FTO	rs11219800	A	0.313276	1.21	0.13	0.93	1.57	0.15
16	CDH13	rs38651880	T	0.303603	1.21	0.13	0.94	1.55	0.13
16	ADIPOQ	rs71937880	G	0.135859	0.85	0.18	0.59	1.23	0.40
16	FTO	rs11076023	T	0.442483	1.05	0.12	0.83	1.33	0.66
16	FTO	rs14210850	C	0.204882	1.17	0.15	0.87	1.57	0.29
16	FTO	rs15589020	A	0.184932	1.22	0.16	0.89	1.65	0.21
16	FTO	rs80501360	A	0.276263	1.15	0.14	0.88	1.52	0.30
16	FTO	rs99305060	G	0.248823	1.23	0.15	0.92	1.64	0.16
18	MC4R	rs22296160	-	0	NA	NA	NA	NA	NA

**Abbreviations**: CHR, chromosome; SNP, single nucleotide polymorphism; MAF, minor allele frequency; OR, odds ratio; SE, standard error.

* Adjusted for sex and age.

**Fig 1:**
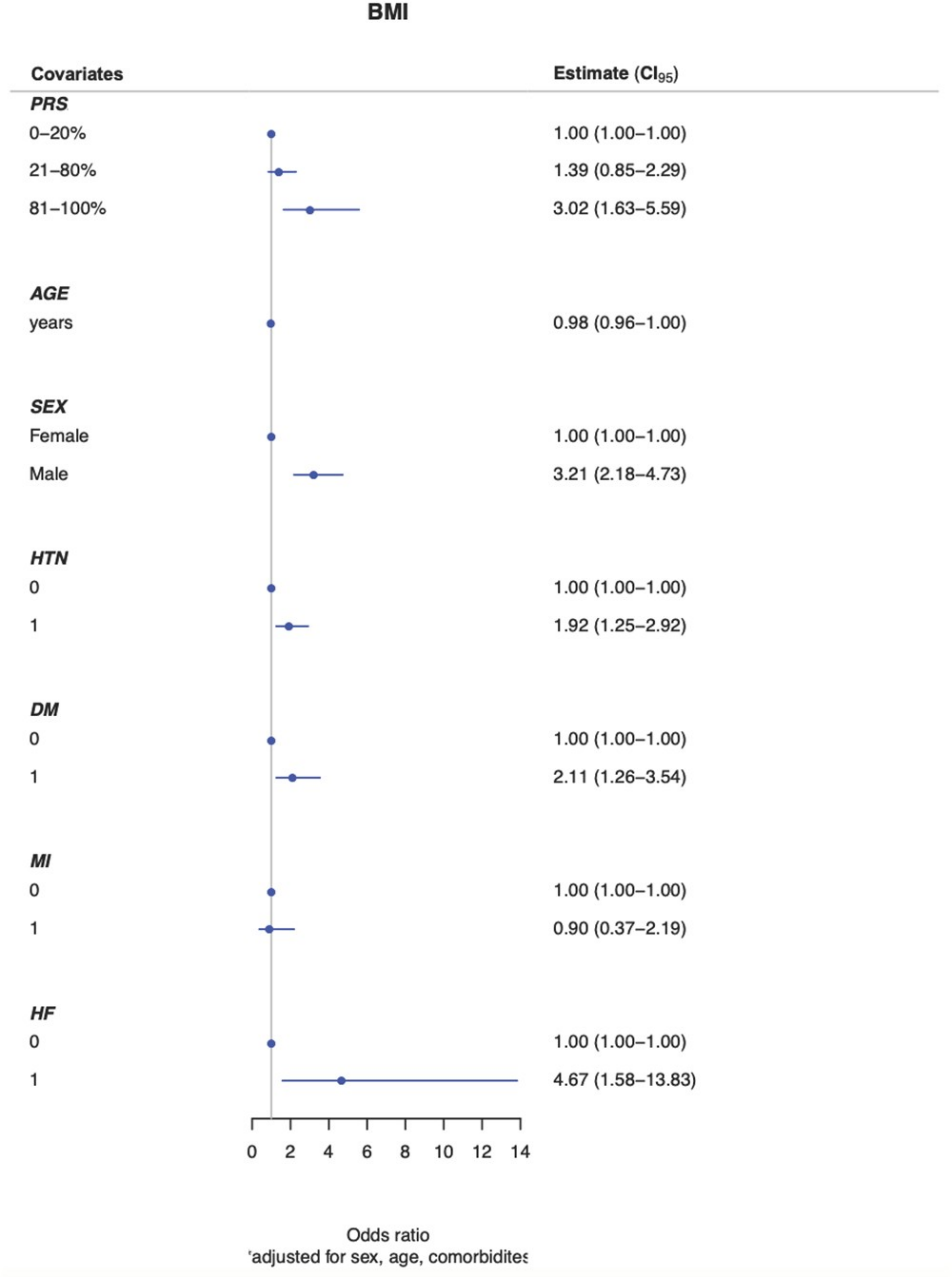
Adjusted polygenic risk score for BMI groups in African Americans. Abbreviations: BMI, body mass index; CI, confidence interval; HTN, hypertension; DM, diabetes mellitus; MI, myocardial infarction; HF, heart failure.

**Fig 2:**
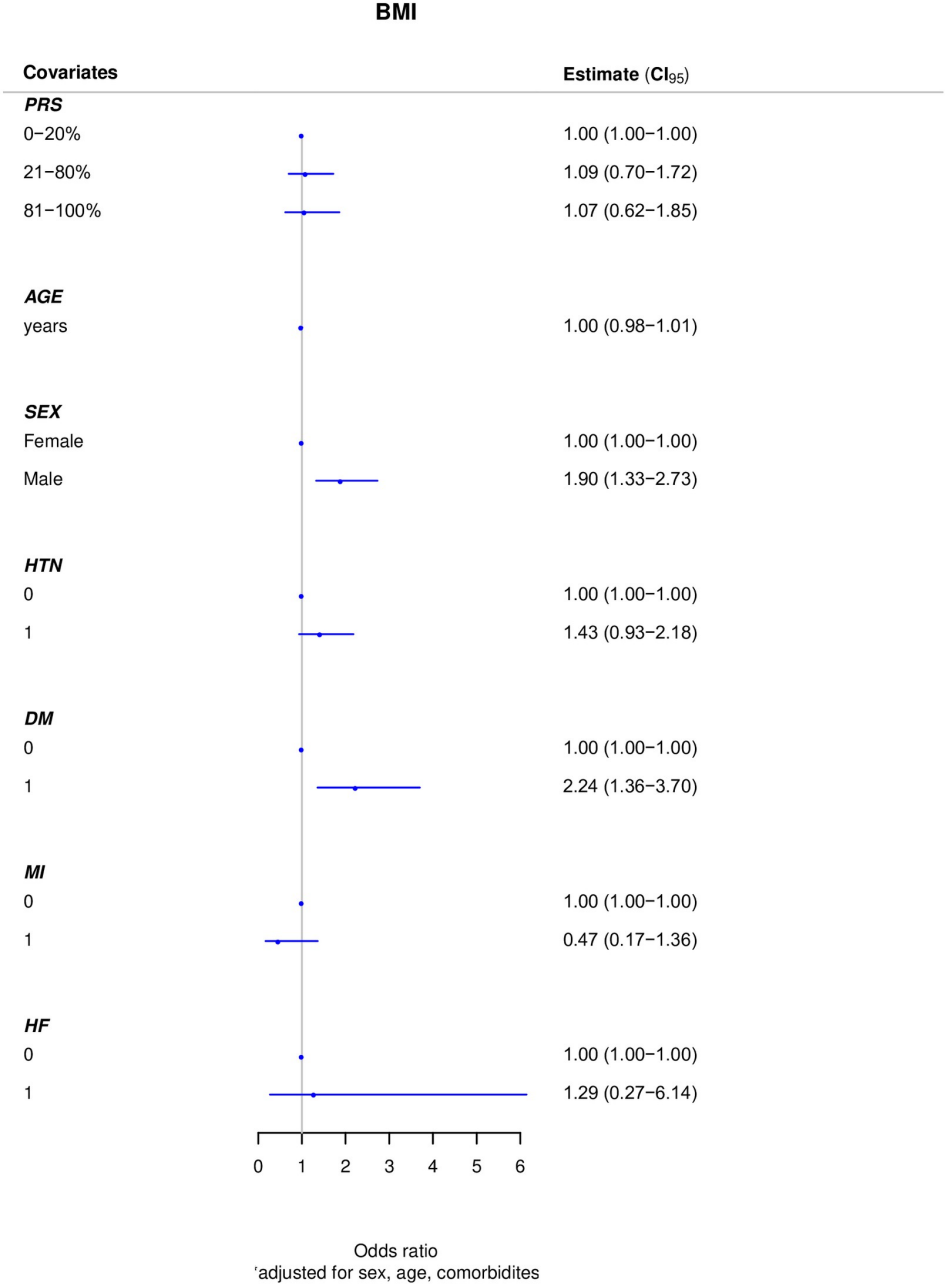
Adjusted polygenic risk score for BMI groups in Hispanics/Latinos. Abbreviations: BMI, body mass index; CI, confidence interval; HTN, hypertension; DM, diabetes mellitus; MI, myocardial infarction; HF, heart failure.

When adding the effects of the common variants, we performed a logistic regression model for known sex, age and clinical risk factors (HTN, DM, MI, CHF) for obesity. In the AA cohort, the intermediate and high genetic risk groups were associated with a 1.39-fold (OR 1.39; 95% [CI]: 0.85–2.29) and 3.02-fold (OR 3.02; 95% [CI]: 1.63–5.59) increase risk in obesity compared to the low genetic risk group. There were no significant differences observed between the different risk categories (intermediate or high compared with low genetic risk) in the H/L cohort (OR 1.09; 95% [CI]: 0.70–1.72) and (OR 1.07; 95% [CI]: 0.62–1.85) respectively (Figs 1 and 2).

## Discussion

The association between obesity risk variants and BMI in ethnic minority populations is poorly understood. Here, we genotyped 26 obesity-associated SNPs previously identified in whites of European descent in AA and H/L individuals. We showed that rs8050136 (in *FTO*) in AAs and rs2272383 (in *TUB*) in H/Ls were associated with obesity. However, none of the SNPs in multivariable analysis of either AA or H/L cohorts were significant when corrected for multiple testing. We also showed that AA individuals with an intermediate or high PRS, were at a three-fold increased risk of developing obesity but this association was not found in the H/L group. Importantly, the *FTO* SNP has previously been reported as a significant contributor to early-onset childhood obesity in whites.

Common genetic variants in the *FTO* locus have consistently been associated with obesity mostly among whites of European descent [12–14]. In 2007, three independent groups identified multiple SNPs in *FTO* located on chromosome 16q12.2 to be strongly associated with obesity in both adults and children of European descent. The strongest signal resided in the first and second intronic regions of *FTO*, which most likely modulates nearby genes [14, 19, 20]. The closest neighboring gene to the *FTO* locus is the *RPGRIP1L* and this is responsible for regulating the trafficking of leptin receptors in cilia to maintain the ciliary function in the body [21, 22]. Defects in this process may lead to obesity, but the underlying mechanisms are poorly understood. A more recent study showed that variants in *FTO* can alter adipocyte function by targeting the *ARID5B*, *IRX3* and *IRX5* thermogenesis pathway [4]. The result of this alteration leads to conversion of the energy-dissipating beige adipocytes to energy-storing white adipocytes, fostering lipid storage and ultimately leading to weight gain.

Some studies have investigated selected *FTO* SNPs in obese individuals from non-European ethnic populations [15, 23]. While several studies in H/L populations replicated the European findings, identifying the causal *FTO* variants in AAs has been more challenging [12, 24–28]. It is thought that this may in part be related to the lower levels of linkage disequilibrium within the variants at the *FTO* locus [29]. A recent meta-analysis identified two novel variants in the *FTO* locus in AAs and this may provide insights into the underlying mechanisms [30, 31].

The *TUB* gene was discovered in the 1990’s as a relevant marker for obesity in mice. It has been hypothesized that this gene, which is highly expressed in the hypothalamus, encodes for a transcription factor that regulates energy homeostasis [32–36]. This process is initiated by the secretion of leptin from adipocyte tissue and binds to receptors in the hypothalamus to inhibit the orexigenic and stimulate the anorexigenic pathways in response to eating food and energy expenditure [37]. Defects in these pathways are postulated to be critical for the obesity phenotype. Despite these advancements, the underlying molecular function of this gene remains unclear. Given the limited number of human studies that have examined the association between *TUB* SNPs and obesity, unraveling the differences in common genetic variation across racial and ethnic groups is important. With improved understanding of the underlying pathophysiologic mechanisms by which *TUB* SNPs increase the risk of obesity will not only provide important insights into the obesity epidemic in the H/L population but also identify potentially therapeutic targets.

Over the last decade, genetic studies have uncovered signaling pathways important in the pathogenesis of obesity. However, translating this knowledge to tackle the obesity epidemic has been limited. Therapeutic targeting of the thermogenesis and orexigenic or anorexigenic pathways that are regulated by the *FTO* and *TUB* genes respectively has important clinical implications for the obesity epidemic especially in ethnic minorities.

This study has a number of limitations that should be addressed. *First*, this was an institutional cohort reflective of a metropolitan population in a large city and our findings may not necessarily reflect rural or other populations where other SNPs may show stronger associations with obesity in minority populations. *Second*, we did not estimate admixture in the obese and non-obese groups. *Third*, there were significantly more women in the obese than non-obese groups most noticeably in AAs and less so in H/Ls. Nonetheless, there remained a significant association between the rs8050136 and rs2272383 SNPs after multiple risk factor adjustments. *Fourth*, several previously reported common SNPs associated with obesity in whites of European decent were not replicated in AAs and H/Ls. This may be in part be explained by the heterogeneity in ethnic minorities and because the linkage disequilibrium structure varies across race and ethnic groups. *Fifth*, we selected SNPs focused on genetic targets thought to be directly associated with obesity and common genetic variation associated with obesity risk factors was not evaluated. Replication of the associations of obesity with *FTO* and *TUB* in independent cohorts is warranted given the limited sample sizes.

## Conclusion

In conclusion, our study confirms the association between common genetic variants in the *FTO* and *TUB* genes and increased risk for developing obesity in individuals of African and Hispanic descent respectively. However, none of the SNPs in multivariable analysis of either AA or H/L cohorts survived correction for multiple testing. Furthermore, implementing a PRS into clinical practice may be considered a useful tool for physicians assessing at-risk individuals. These findings highlight the importance of genetic variation in the pathogenesis of obesity in ethnic minority populations in order to understand individualized genetic risk factors for obesity, both in terms of more accurate risk stratification and mechanistic studies. Further validation and functional studies are needed to confirm our findings and to determine the underlying mechanisms by which *FTO* and *TUB* SNPs modulate susceptibility to obesity across racial and ethnic groups.
